# Environmental and Genetic Determinants of Biofilm Formation in Paracoccus denitrificans

**DOI:** 10.1128/mSphereDirect.00350-17

**Published:** 2017-09-06

**Authors:** Santosh Kumar, Stephen Spiro

**Affiliations:** Department of Biological Sciences, University of Texas at Dallas, Richardson, Texas, USA; University of Wisconsin—Madison; Stony Brook University; University of East Anglia

**Keywords:** biofilm, cyclic di-GMP, denitrification, flavohemoglobin, H-NOX, nitric oxide, Paracoccus denitrificans

## Abstract

The genome of the denitrifying bacterium Paracoccus denitrificans predicts the expression of a small heme-containing nitric oxide (NO) binding protein, H-NOX. The genome organization and prior work in other bacteria suggest that H-NOX interacts with a diguanylate cyclase that cyclizes GTP to make cyclic di-GMP (cdGMP). Since cdGMP frequently regulates attached growth as a biofilm, we first established conditions for biofilm development by *P. denitrificans*. We found that adhesion to a polystyrene surface is strongly stimulated by the addition of 10 mM Ca^2+^ to rich media. The genome encodes at least 11 repeats-in-toxin family proteins that are predicted to be secreted by the type I secretion system (TISS). We deleted the genes encoding the TISS and found that the mutant is almost completely deficient for attached growth. Adjacent to the TISS genes there is a potential open reading frame encoding a 2,211-residue protein with 891 Asp-Ala repeats. This protein is also predicted to bind calcium and to be a TISS substrate, and a mutant specifically lacking this protein is deficient in biofilm formation. By analysis of mutants and promoter reporter fusions, we show that biofilm formation is stimulated by NO generated endogenously by the respiratory reduction of nitrite. A mutant lacking both predicted diguanylate cyclases encoded in the genome overproduces biofilm, implying that cdGMP is a negative regulator of attached growth. Our data are consistent with a model in which there are H-NOX-dependent and -independent pathways by which NO stimulates biofilm formation.

**IMPORTANCE** The bacterium Paracoccus denitrificans is a model for the process of denitrification, by which nitrate is reduced to dinitrogen during anaerobic growth. Denitrification is important for soil fertility and greenhouse gas emission and in waste and water treatment processes. The ability of bacteria to grow as a biofilm attached to a solid surface is important in many different contexts. In this paper, we report that attached growth of *P. denitrificans* is stimulated by nitric oxide, an intermediate in the denitrification pathway. We also show that calcium ions stimulate attached growth, and we identify a large calcium binding protein that is required for growth on a polystyrene surface. We identify components of a signaling pathway through which nitric oxide may regulate biofilm formation. Our results point to an intimate link between metabolic processes and the ability of *P. denitrificans* to grow attached to a surface.

## Abstract (precis)

The bacterium Paracoccus denitrificans is a model for the process of denitrification, by which nitrate is reduced to dinitrogen during anaerobic growth. Denitrification is important for soil fertility and greenhouse gas emission and in waste and water treatment processes. The ability of bacteria to grow as a biofilm attached to a solid surface is important in many different contexts. In this paper, we report that attached growth of *P. denitrificans* is stimulated by nitric oxide, an intermediate in the denitrification pathway. We also show that calcium ions stimulate attached growth, and we identify a large calcium binding protein that is required for growth on a polystyrene surface. We identify components of a signaling pathway through which nitric oxide may regulate biofilm formation. Our results point to an intimate link between metabolic processes and the ability of *P. denitrificans* to grow attached to a surface.

## INTRODUCTION

Paracoccus denitrificans is a metabolically versatile alphaproteobacterium that has been extensively used as a model for studies of oxidative phosphorylation, anaerobic respiration (denitrification), and the metabolism of one-carbon compounds (1). Typically described as soil bacteria, *Paracoccus* spp. in fact seem rather ubiquitous, being also found in aquatic environments (2), waste treatment processes (3), and the deep subsurface (4) and in association with plants (5) and animals (6). *P. denitrificans* is a facultative anaerobe and an obligatorily respiratory organism. Under anoxic growth conditions, substrate oxidation is coupled to the sequential reduction of nitrogen oxyanions and oxides to dinitrogen, in the pathway called denitrification. Denitrification is an important route for the loss of fixed nitrogen from soil and is applied in water and waste treatment processes. In both cases, denitrification attracts interest as a significant source of the greenhouse gas nitrous oxide (7).

Nitric oxide (NO) metabolism by *P. denitrificans* is thought to depend principally upon a respiratory membrane-associated NO reductase, NOR (or NorBC), which couples oxidation of cytochrome *c* to the reduction of NO to nitrous oxide (N_2_O). Expression of the *nor* operon is activated in response to NO by the transcriptional regulator NNR, which also activates genes encoding the respiratory nitrite reductase NirS that reduces nitrite to NO (8, 9). Thus, NO is a key regulatory signal controlling the transition from aerobic to anaerobic metabolism (10, 11).

The *P. denitrificans* genome reveals a rich repertoire of unstudied genes and proteins that potentially mediate additional responses to NO (Table 1). There are two homologs of the NO-sensitive repressor NsrR (12, 13). On the basis of genome context, one (Pden_1690) is predicted to regulate expression of the *hmp* gene (Pden_1689) encoding an NO-scavenging flavohemoglobin (14, 15), together with a closely linked gene of unknown function (Pden_1688). The second NsrR (Pden_3024) is predicted to regulate expression of *ytfE*, which encodes a protein from the RIC family that is involved in the repair of NO-damaged [Fe-S] proteins (16). Additionally, the genome encodes H-NOX (Pden_3719), a small NO binding heme protein (17–19). Biological roles of H-NOX proteins have not been well studied, but there are examples involved in the regulation of iron metabolism (20) and of motility versus attached growth (18, 21). The *P. denitrificans* H-NOX is encoded by a gene that overlaps the gene (Pden_3720) encoding a protein with a GGDEF domain. The GGDEF domain is associated with a diguanylate cyclase activity that cyclizes GTP to form cyclic di-GMP (cdGMP), a second messenger involved in the regulation of many bacterial processes (22). The *P. denitrificans* genome organization suggests that NO has the potential to regulate cdGMP synthesis and downstream processes, as is the case in some other bacteria (18, 19). In Shewanella woodyi, H-NOX interacts with a bifunctional diguanylate cyclase/cdGMP phosphodiesterase (23). In this case, the NO-bound form of H-NOX stimulates phosphodiesterase activity to lower the cytosolic concentration of cdGMP, an event which downregulates biofilm formation (24).

**TABLE 1:** Paracoccus denitrificans genes discussed in this paper

Gene no.	Gene name	Function
Pden_0876	*pdeA*	cdGMP phosphodiesterase (predicted to be inactive)
Pden_1689	*hmp* (*fhp*)	NO denitrosylase
Pden_1690	*nsrR1*	NO-sensitive repressor NsrR
Pden_2025	*pdeB*	cdGMP phosphodiesterase
Pden_2411	*bapD*	TISS outer membrane protein
Pden_2412	*bapC*	TISS membrane fusion protein
Pden_2413	*bapB*	TISS ABC transporter
n/a^a^	*bapA*	TISS-secreted RTX family agglutinin
Pden_3024	*nsrR2*	NO-sensitive repressor NsrR
Pden_3025	*ytfE*	RIC family [Fe-S] repair protein
Pden_3719	*hnoX*	H-NOX protein
Pden_3720	*dgcA*	Diguanylate cyclase
Pden_3982	*dgcB*	Diguanylate cyclase (fused to response regulator domain)

a n/a, the *bapA* gene is not present in the original annotation of the *P. denitrificans* genome and so does not have a Pden gene number. The reading frame is present in the manually curated SEED annotation (http://rast.nmpdr.org/seedviewer.cgi?page=Annotation&feature=fig|318586.4.peg.2260) and has been described by other authors (41).

Biofilm formation and motility have been described as being reciprocally regulated by cdGMP in several species. Typically, cdGMP inhibits motility and stimulates attached growth as a biofilm (22). *P. denitrificans* is a nonmotile bacterium and lacks most (though not all) genes that are associated with flagellum-based motility and chemotaxis. The genetic basis of biofilm formation by this organism has not been well studied but is a potential target for regulation by cdGMP. In biological water and sewage treatment processes, microorganisms frequently grow as surface-attached biofilms or as granules, and *Paracoccus* spp. have been observed in both cases (25, 26), suggesting that it is capable of attached growth. It is important to understand how metabolism in biofilms contributes to the efficiency of chemical transformations in treatment processes. In other bacteria, respiratory metabolism and electron acceptor availability are intimately linked to biofilm development (27).

In this paper, we report conditions that allow robust and reproducible biofilm formation by *P. denitrificans*. We show that attached growth is stimulated by calcium ions and suggest that attachment requires one or more calcium binding proteins secreted to the cell envelope through a type I secretion system. We present evidence to suggest that endogenously generated NO is a signal that stimulates attached growth and that the H-NOX/cdGMP signaling pathway has a role in the regulation of NO metabolism and biofilm formation.

## RESULTS

### Biofilm formation by *P. denitrificans*.

**FIG 1:**
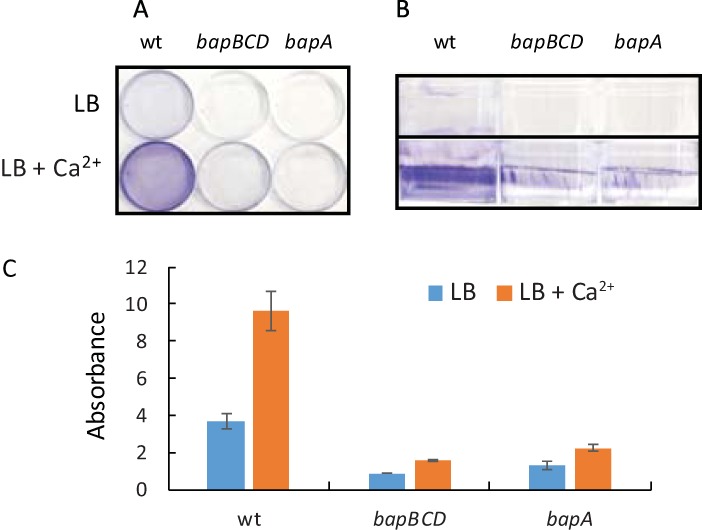
Assays of biofilm formation by *P. denitrificans* Pd1222 and by Δ*bapBCD* and Δ*bapA* mutants. Cultures were incubated for 72 h without shaking in 6-cm-diameter petri dishes. The growth medium was liquid L broth, supplemented with 10 mM calcium chloride as indicated. (A and B) Dishes were washed, stained with crystal violet, and photographed from the top (A) and side (B). (C) Crystal violet was extracted from biofilms and quantified by absorption at 595 nm. Means and standard deviations were calculated from results of triplicate assays. wt, wild type.

We established conditions for biofilm formation in *P. denitrificans* and then assayed the phenotypes of mutants and the influence of environmental conditions. We found that *P. denitrificans* can form adhered biofilms when standing cultures are grown in rich medium in conventional polystyrene petri dishes. After 3 to 4 days of incubation, attached growth is detectable on the bottom surface of the petri dish (Fig. 1A), in addition to being detectable as a prominent ring that forms at the air-liquid interface (Fig. 1B). Bottom-attached growth is dependent on the surface area of the medium, being denser in 9-cm-diameter petri dishes than in 6-cm-diameter petri dishes and absent in 1-cm-diameter 24-well microplates (not shown). The effect of surface area and the growth at the air-liquid interface suggest that oxygen availability is a determinant of biofilm formation in *P. denitrificans*. Biofilm formation at the air-liquid interface occurs commonly, and this behavior has been observed in Pseudomonas fluorescens and Escherichia coli (28, 29), among other bacterial species.

### Identification of a secretion pathway and potential adhesin required for attached growth.

Sessile attached cells typically grow in a matrix that is formed by extracellular polysaccharide, nucleic acid, or protein. Information in the KEGG database (30) provides no evidence of an ability of *P. denitrificans* to synthesize extracellular polysaccharide. The genome annotation suggests that glycogen is the only polysaccharide made by this organism. We therefore searched for evidence of extracellular proteins that might function as adhesins. We used PSORTdb (31) to retrieve predictions of the subcellular location of the *P. denitrificans* proteome. This analysis identified 11 extracellular or cell envelope-associated proteins from the repeats-in-toxin (RTX) family (see Fig. S1 in the supplemental material). These proteins of diverse function are characterized by the presence of multiple nonapeptide calcium binding domains and secretion by the type I secretion system (32). The RTX family includes proteins that function as adhesins (33). In 3-day incubations, we found that the addition of 10 mM Ca^2+^ to growth media strongly stimulated biofilm formation (Fig. 1). These observations are consistent with the idea of attached growth depending on extracellular Ca^2+^ binding proteins. The calcium ion concentration of a rich medium similar to ours has been estimated to be 120 μM (34). Our defined medium formulation contains 0.68 mM calcium chloride and 19.6 μM EDTA (35). Compared to the results seen with rich medium, we observed more attached growth in unsupplemented minimal medium (consistent with the higher calcium concentration) and so a lower level of stimulation by additional calcium (data not shown). Calcium supplementation was also prone to causing precipitation in minimal medium. Therefore, all cultures discussed here were grown in rich medium in 6-cm-diameter petri dishes.

10.1128/mSphereDirect.00350-17.1 FIG S1 Proteins encoded in the Paracoccus denitrificans genome from the repeats-in-toxin (RTX) family. Domain annotations are from Pfam (pfam.xfam.org). Short unlabeled motifs (colored green or red) are hemolysin-type calcium binding domains. The number of amino acid residues in each protein is shown. Download FIG S1, EPS file, 2.2 MB. Copyright © 2017 Kumar and Spiro. 2017 Kumar and Spiro This content is distributed under the terms of the Creative Commons Attribution 4.0 International license.

We analyzed the *P. denitrificans* proteome using the TXSScan algorithm (36), which revealed the presence of a single predicted type I secretion system (TISS) encoded by Pden_2411 to Pden_2413 (Table 1). These genes encode the ABC transporter (Pden_2413), membrane fusion protein (Pden_2412), and outer membrane efflux protein (Pden_2411) components of the TISS (37). We designate these genes *bapBCD*, for reasons explained below. To generate a null TISS-deficient mutant, we deleted these three genes. The resultant Δ*bapBCD* strain had no gross growth defect in liquid culture or on agar (not shown) but was almost completely deficient for biofilm formation on polystyrene (Fig. 1). Our minimal interpretation of these results is that attached growth in *P. denitrificans* requires one or more of the extracellular Ca^2+^ binding proteins that are secreted by the TISS pathway.

**FIG 2:**
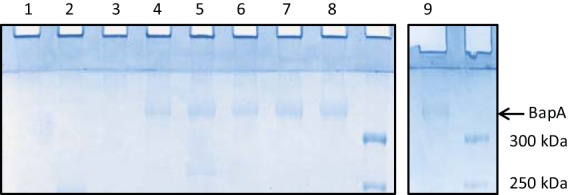
Detection of BapA in envelope fractions of *P. denitrificans*. Planktonic phase cells were harvested from biofilm assays (grown in liquid L broth supplemented with 10 mM calcium chloride), and envelope fractions were prepared and separated by SDS-PAGE. The protein indicated by the arrow was shown by mass spectrometry to be BapA. The strains analyzed were as follows: lane 1, Δ*bapBCD* mutant; lane 2, wild type; lane 3, Δ*hnoX* mutant; lane 4, Δ*dgcA* mutant; lane 5, Δ*dgcB* mutant; lane 6, Δ*dgcA* Δ*dgcB* mutant; lane 7, Δ*hmp* mutant; lane 8, Δ*hmp* Δ*hnoX* mutant; lane 9, Δ*nor* mutant. Two gel images are shown since the number of samples exceeded the capacity of the gel apparatus.

To search for direct evidence of extracellular adhesins, cells from the planktonic phase of biofilm assays (grown in rich medium supplemented with 10 mM Ca^2+^) were fractionated to enrich for periplasmic and outer membrane proteins (38), which were then separated by denaturing polyacrylamide gel electrophoresis (SDS-PAGE). A very large (>300-kDa) protein was visible in envelope fractions (Fig. 2) and had increased abundance in mutants with hyperbiofilm phenotypes (see below). The accumulation of this protein correlates very well with genotypes that stimulate biofilm accumulation, which, together with its size and location, makes it a good candidate for an adhesin.

In the manually curated SEED (39) annotation of the *P. denitrificans* genome, there is a 6,636-bp gene immediately upstream of the three genes encoding the TISS (http://rast.nmpdr.org/seedviewer.cgi?page=Annotation&feature=fig|318586.4.peg.2260). The 2,211-amino-acid product of this gene (designated *bapA*, as explained below) is annotated as a TISS-secreted RTX family agglutinin. A total of 1,782 residues of the BapA protein consist of 891 repeats of the dipeptide Asp-Ala, with divergent sequences at the N and C termini. The PSORTdb algorithm (31) strongly predicts BapA to be located in the outer membrane (it was absent from our original analysis because this open reading frame is annotated only in the SEED database). The BapA protein has a molecular mass of 212 kDa and a predicted pI of 2.4. Mass spectrometry (MS) of excised gel slices confirmed that the largest envelope protein detected by SDS-PAGE is BapA. In two independent samples, BapA was identified as the most abundant protein in the excised band by normalized spectral counts and the spectral index criterion (40). All BapA tryptic peptides below 40 amino acids in length were detected, and BapA was identified with 134 peptide spectrum matches and 10 unique peptide sequences, with a false-discovery rate of 1%. Thus, we have confidence in the assignment of the stained band in envelope fractions (Fig. 2) as BapA. The BapA protein has an apparent molecular weight in SDS-PAGE that is significantly higher than its true molecular weight (Fig. 2). This discrepancy may relate to the highly unusual amino acid composition of BapA (41.8% alanine, 42.6% aspartate), although we cannot exclude the possibility of a posttranslational modification. We constructed an unmarked deletion in the *bapA* gene and found that the mutant has a phenotype similar to that of the stain lacking the TISS, that is, a severe defect in biofilm formation (Fig. 1). BapA is almost undetectable on Coomassie-stained gels of the envelope fraction of a wild-type strain (Fig. 2, lane 2). Nevertheless, we assume that the wild-type strain makes sufficient BapA to promote the degree of attached growth visible in petri dishes.

To summarize, we have shown that biofilm formation by *P. denitrificans* is stimulated by calcium ions and depends upon a functional TISS. Genetic evidence implicates a large envelope protein that is a predicted TISS substrate as being required for attached growth, perhaps functioning as an adhesin. We cannot exclude the possibility that other TISS substrates (of which there are at least 11 predicted) also have roles in attached growth.

As we were completing this work, similar conclusions about the role of the *P. denitrificans* TISS and the RTX family agglutinin were reported elsewhere (41). The three genes encoding the components of the TISS were designated *bapBCD*, and the gene encoding the secreted adhesion was designated *bapA*; we have therefore adopted this nomenclature (Table 1). Our work extends the findings of Yoshida et al. (41) by demonstrating a clear role for calcium ions in attached growth, by detecting BapA directly by SDS-PAGE, and by showing that BapA abundance correlates, at least qualitatively, with the extent of attached growth (see below).

### Endogenously generated nitric oxide stimulates biofilm formation.

**FIG 3:**
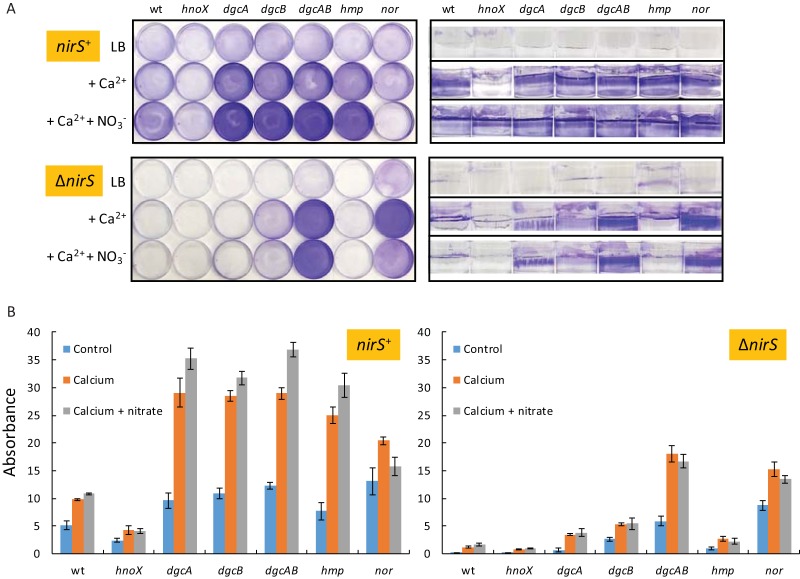
Assays of biofilm formation by *P. denitrificans* Pd1222 and Δ*hnoX*, Δ*dgcA*, Δ*dgcB*, Δ*dgcAB*, Δ*hmp*, and Δ*nor* mutants. Cultures were incubated for 72 h without shaking in 6-cm-diameter petri dishes. The growth medium was L broth, supplemented with 10 mM calcium chloride and 5 mM nitrate as indicated. (A) Dishes were washed, stained with crystal violet, and photographed from the top and side. (B) The crystal violet was extracted with ethanol, and the absorbance was measured at 595 nm (68). Means and standard deviations were calculated from results of triplicate assays. The strains used were either wild type (*nirS*^+^) or deleted (Δ*nirS*) for the nitrite reductase gene *nirS* as indicated.

Recent work has shown that the flavohemoglobin of *P. denitrificans* protects against extracellular nitrite during aerobic growth, presumably because of its ability to scavenge traces of NO generated abiotically from nitrous acid (42). We have constructed an *hmp* mutant and confirmed that it metabolizes NO more slowly than the wild type in whole-cell assays of NO consumption (data not shown). Surprisingly, the *hmp* mutant also overproduces biofilm compared to a wild-type strain (Fig. 3). We have previously shown that our formulation of L broth contains approximately 0.5 mM nitrate which can act as a source of endogenously generated NO under oxygen-limiting conditions (43). To explain the phenotype of the *hmp* mutant, we hypothesize that oxygen limitation in these static cultures initiates expression of denitrification genes. Thus, nitrate is reduced to nitrite and nitrite to NO. Since the *hmp* mutant is lacking one pathway for NO consumption, elevated NO concentrations in this strain might explain the increase in biofilm, if NO is a signal that stimulates biofilm formation. To test this hypothesis, we first grew static biofilm cultures of strains containing *hmp-lacZ* and *norC-lacZ* reporter fusions. The *norC* promoter is activated by the NO-sensing transcription factor NNR (44), and the *hmp* promoter is predicted to be subject to negative regulation by the NO-sensitive repressor NsrR1. In static cultures in unsupplemented medium, the *hmp* promoter has activity as high as that measured in cultures grown in shake flasks and treated with a source of NO (Table 2). In contrast, the *norC* promoter has a rather low and submaximal level of activity in unsupplemented static cultures. Therefore, we conclude that NO is generated endogenously in static cultures to an extent that efficiently induces the *hmp* but not the *norC* promoter.

**TABLE 2:** Activities of promoter reporter fusions measured in shake flasks and in static cultures

Reporter fusion	β-Galactosidase activity (mean ± SD)^a^			
	Shake flask	Shake flask + NO	Static	Static + 5 mM nitrate
*hmp-lacZ*	391 ± 12	824 ± 12	895 ± 40	1,205 ± 109
*norC-lacZ*	91 ± 5	329 ± 17	179 ± 5	2,103 ± 140

a Liquid cultures were grown in shake flasks to mid-log phase or for 72 h in static 6-cm-diameter petri dishes (the same conditions used for biofilm assays). For NO-treatment, shake flask cultures were amended with 50 μM spermine NONOate 60 min prior to sampling. β-Galactosidase was assayed according to Miller (69). Means and standard deviations were calculated from results of triplicate assays.

To confirm the source of the endogenously generated NO and that it stimulates biofilm formation, we introduced a *nirS* mutation into the previously characterized strains. Mutation of *nirS* abolishes biofilm formation in both the wild-type strain and the *hmp* mutant (Fig. 3), which strongly suggests that reduction of nitrite to NO generates a signal that stimulates biofilm formation. It is unclear why the Δ*nirS* Δ*nor* double mutant overproduces biofilm compared to the Δ*nirS* strain (Fig. 3).

The abundance of BapA is higher in the *hmp* mutant than in the wild-type strain (Fig. 2, lane 7), which may explain the hyperbiofilm phenotype of the *hmp* mutant. An interesting possibility is that elevated NO levels in the *hmp* mutant might lead (either directly or indirectly) to increased expression of the *bapA* gene.

### Regulation of biofilm formation by cdGMP.

**FIG 4:**
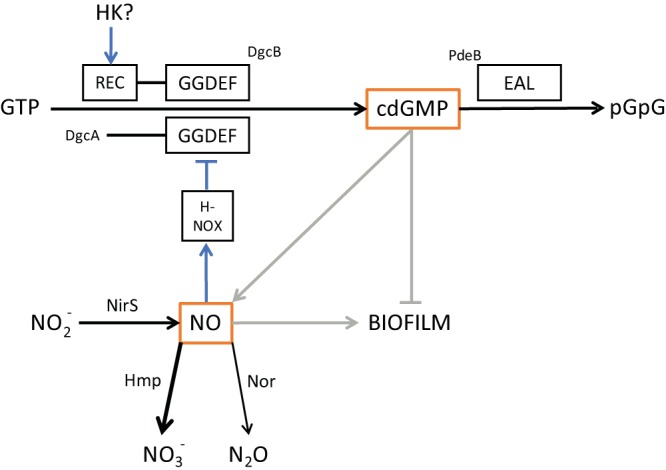
A model for the regulation of biofilm formation in Paracoccus denitrificans based on the work described in this paper. Abbreviations: H-NOX, heme NO and oxygen binding; REC, receiver domain; HK, histidine kinase. GGDEF and EAL are sequence motifs associated with the diguanylate cyclase and phosphodiesterase, respectively. Enzymes and enzyme-catalyzed reactions are shown in black, signaling interactions in blue and gray (the latter indicating pathways that are likely to be indirect). We suggest that Hmp is the dominant pathway for NO metabolism in the static cultures used for biofilm assays.

According to the MIST database (45), the *P. denitrificans* genome encodes two proteins with GGDEF domains associated with the synthesis of cdGMP (Fig. 4). One (Pden_3720; DgcA) has an N-terminal domain of unknown function, and the product of the adjacent Pden_3719 gene is an H-NOX protein (18). Like the H-NOX from its close relative Rhodobacter sphaeroides (17), the *P. denitrificans* H-NOX lacks the otherwise conserved histidine residue which functions as the axial ligand to the heme (18). The genome organization suggests that H-NOX regulates the biosynthesis of cdGMP. There is a similar situation in Legionella pneumophila and Shewanella woodyi, though in these cases the H-NOX protein regulates the activities of a bifunctional enzyme that both synthesizes and degrades cdGMP (24, 46). The second GGDEF domain-containing protein (Pden_3982; DgcB) has an N-terminal receiver domain, implying a signal input pathway from an unidentified histidine kinase. The *P. denitrificans* genome encodes two proteins with EAL domains associated with cdGMP hydrolysis (Fig. 4), but one of these is predicted to be catalytically inactive (47). Catalytically inactive EAL domains can potentially function as cdGMP receptors (22).

**FIG 5:**
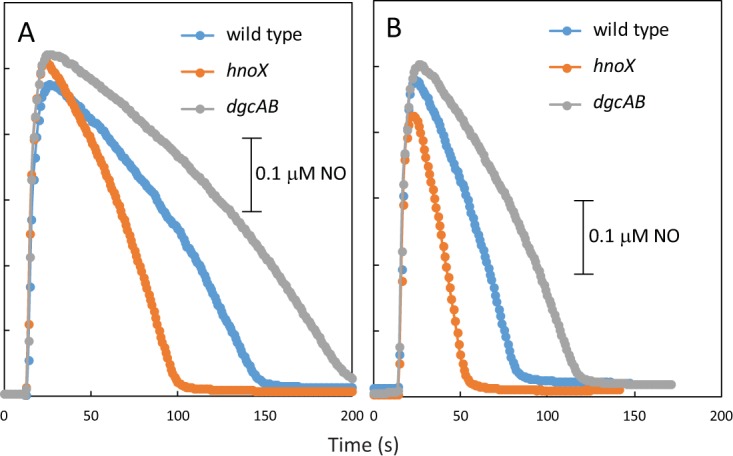
Assays of NO consumption by washed cell suspensions of *P. denitrificans* and its *hnoX* and *dgcAB* mutants. Cultures were grown aerobically in succinate minimal media (A) and were treated with 50 μM spermine NONOate for 60 min prior to harvesting (B) in order to induce expression of the *hmp* and *nor* genes.

To begin to explore the function of the cdGMP signaling pathway in *P. denitrificans*, we have characterized mutants lacking single and multiple genes encoding pathway components. In assays of nitric oxide (NO) consumption by washed cell suspensions, we found that the cdGMP-deficient mutant (Δ*dgcAB*) consumes NO somewhat more slowly than the wild-type parent and that the *hnoX* mutant consumes NO more rapidly (Fig. 5). Reporter fusion assays showed no effects of *hnoX* and *dgcAB* mutations on *norC* or *hmp* promoter activity (data not shown). Therefore, it is likely that cdGMP regulation of NO consumption does not operate at the level of gene expression.

In biofilm assays, the *hnoX* mutant is partially deficient for attached growth, while the *dgcAB* and *hmp* mutants overproduce biofilm (Fig. 3). The BapA protein overaccumulates in the same strains (mutants Δ*hmp* and Δ*dgcAB*) that overproduce biofilm and is absent in the *hnoX* mutant (Fig. 2, lanes 7, 6, and 3, respectively), further evidence of a correlation between biofilm density and BapA abundance, which is consistent with this protein having a function in attached growth. The data also suggest that *bapA* expression or the secretion of the BapA protein to the cell envelope (or both) is regulated by cdGMP and NO.

The overproduction of biofilm by the *dgcAB* mutant is consistent with the possibility that cdGMP regulates biofilm formation and/or that NO accumulates in this mutant (because of the reduced rate of consumption) and stimulates biofilm formation as described above. To distinguish these possibilities, we introduced the Δ*nirS* mutation into the *dgcAB* mutant. The *dgcAB nirS* mutant continued to overproduce biofilm compared to the Δ*nirS* parent (Fig. 3). This observation implies that the increased-biofilm phenotype of the *dgcAB* mutant cannot be ascribed solely to accumulation of NO and that cdGMP may therefore negatively regulate biofilm formation independently of NO (Fig. 4).

## DISCUSSION

Large cell surface proteins that have roles in the biofilm matrix have been identified in multiple species (33, 48–50). In many cases, these proteins are characterized by the occurrence of complex and/or simple repeat sequences. One such protein that is quite well understood and that may serve as a model for BapA is SdrC of Staphylococcus aureus (51). SdrC is a 995-residue multidomain protein with 109 Ser-Asp repeats. SdrC is involved in cell-cell and cell-surface interactions, and these appear to be separable functions of the protein. Homophilic SdrC-mediated cell-cell interactions require two pentapeptide sequences, RPGSV and VDQYT, separated by 37 residues (51). Interestingly, there are very similar sequences (LPGSV and VDRYT) in the *P. denitrificans* BapA protein, although the spacing is much greater, since these motifs occur on either side of the Asp-Ala repeats. SdrC also promotes adhesion to plastic surfaces (51); this is a strong interaction believed to involve the unfolding of SdrC, exposing hydrophobic residues responsible for the protein-surface adhesion (52). The N- and C-terminal domains of BapA that flank the Asp-Ala repeats are quite hydrophobic (being 26% and 22% aliphatic residues, respectively) and have sequence similarity to other adhesins (41). Thus, it is possible that BapA mediates both cell-cell and cell-surface interactions in a manner similar to that seen withSdrC. This idea is consistent with the observation that BapA contributes to cell surface hydrophobicity (41), although the suggestion that the Asp-Ala repeats contribute directly to hydrophobicity (41) does not seem intuitively reasonable. The function of the Ser-Asp repeats of SdrC is not known; nor is the role of metal ions in SdrC function fully understood. SdrC-mediated biofilm formation is inhibited by manganese ions but is not affected by calcium (51).

The primary structure of BapA and the stimulatory effect of calcium ions suggest other possible mechanisms for BapA activity. Extracellular aspartate-rich proteins that bind calcium ions have been documented in mammals (53, 54). Aspartate-rich proteins are also found in the shell matrices of bivalves such as oysters. Aspein is one such protein and is characterized by extreme aspartate richness and the presence of Asp-Ala repeat sequences (55). Cell surface carboxylates may function to nucleate the precipitation of calcium carbonate (56), and aspein and other polyanionic proteins are believed to play a role in the formation of the calcite mineral component of the shell (57). Bacteria are well known to mineralize calcium, and denitrification is one of the metabolic processes associated with calcium carbonate formation (58). Calcium carbonate has been detected in bacterial biofilms, for example, in the case of Pseudomonas aeruginosa (59); thus, we speculate that BapA might function to nucleate the formation of a calcium mineral that is required for cell-cell and cell-surface interactions. An alternative possibility is that binding of calcium ions to BapA alters the structure of the protein in a way that is required for it to function as an adhesin. This mechanism operates for some other large cell surface calcium-dependent adhesins, such as LapF of Pseudomonas fluorescens (60).

Our mutant analysis suggests a role for the H-NOX pathway and cdGMP in the regulation of biofilm formation (Fig. 4). In contrast to most other bacteria (22), cdGMP acts negatively on biofilm, and this can, at least in part, be attributed to regulation of BapA abundance (Fig. 1 and 2). We suggest that H-NOX inhibits the activity of DgcA, since the *hnoX* mutant underproduces both biofilm and BapA (Fig. 2 and 3). This implies that NO stimulates H-NOX-mediated inhibition of DgcA to reduce the size of the pool of cdGMP and so to stimulate biofilm formation. Given that NirS-derived NO stimulates biofilm in *hnoX* and *dgc* mutants (Fig. 3) and that an *hnoX hmp* double mutant forms biofilm and overproduces BapA like an *hmp* mutant (Fig. 2 and data not shown), we believe that NO can also stimulate biofilm formation independently of H-NOX/DgcA (Fig. 4).

## MATERIALS AND METHODS

### Bacterial strains and growth conditions.

All *P. denitrificans* mutants were derived from strain Pd1222 (61). Escherichia coli S17-1 (62) was used as the donor for the introduction of plasmids into *P. denitrificans* by conjugation (63). E. coli Novablue Singles competent cells (Novagen) were used as the host for routine DNA manipulations. The rich medium was L broth (10 g tryptone·liter^−1^, 5 g yeast extract·liter^−1^, 10 g NaCl·liter^−1^), and the defined medium for *P. denitrificans* was as previously described (35). Bacteria were cultured at 37°C (E. coli) or 30°C (*P. denitrificans*).

### Genetic manipulations.

Unmarked deletions were made by allelic replacement using pK18*mobsacB* (64) and the method described by Sullivan et al. (65). Briefly, in-frame unmarked deletions were constructed by PCR amplification of ~600-bp fragments from each side of the gene of interest. The two PCR amplicons were ligated together by PCR using overlapping regions added in the primers (66). The ligated fragment was cloned into pK18*mobsacB*, transformed into E. coli S17-1 and then mobilized into *P. denitrificans* by conjugation. Single-crossover events were selected on kanamycin plates, and then recombinants with double crossovers were isolated on the basis of sucrose resistance and kanamycin sensitivity. The structures of chromosomal deletions were confirmed by PCR. Promoter-*lacZ* reporter fusions were constructed by cloning ~400 bp upstream of the start codon of genes of interest into pMP220 (67). Plasmid clones were introduced into *P. denitrificans* by conjugation from E. coli S17-1. Triparental matings were performed by mixing log-phase cells of *P. denitrificans*, E. coli S17-1 cells harboring recombinant pMP220 or pK18*mobsacB* derivatives, and E. coli DH5α (pRK2013) cells in a 2:1:1 ratio and by incubation on L agar for 24 h at 30°C. *P. denitrificans* exconjugants were selected by analysis of rifampin or tetracycline resistance. Plasmid pSTBlue-1 (Novagen) was used as the vector for routine DNA manipulations and sequencing.

### Biofilm assays.

Attached growth was assayed in 60-mm-by-15-mm polystyrene petri dishes. A 100-μl aliquot of overnight culture was inoculated into 7 ml of L broth amended with calcium chloride as indicated. The petri dishes were incubated for about 72 h at 30°C without shaking. The plates were then washed three times with water and treated with crystal violet (0.1% in water) for 20 min, washed three times with water, and air dried. For quantitation, the stain was extracted into 2 ml 95% ethanol and diluted as necessary, and the absorbance was measured at 595 nm (68).

### Biochemical and other methods.

Envelope proteins were enriched by cell fractionation (38) and resolved by denaturing gel electrophoresis using 5% polyacrylamide. Stained bands of interest were excised from gels and analyzed by mass spectrometry in the Proteomics Facility at the University of Texas Southwestern Medical School. Tandem mass spectrometry (MS/MS) spectra were collected for tryptic fragments, and data were analyzed with an in-house pipeline, with quantitation according to the normalized spectral index method (40). Beta-galactosidase activity was measured in cells made permeable by treatment with chloroform and SDS (69). Nitric oxide consumption by washed cell suspensions (normalized according to optical density) was measured with an amperometric NO-specific electrode (ISO NOP electrode; WPI Instruments) as described previously (70).
